# Discrimination of Timbre in Early Auditory Responses of the Human Brain

**DOI:** 10.1371/journal.pone.0024959

**Published:** 2011-09-15

**Authors:** Jaeho Seol, MiAe Oh, June Sic Kim, Seung-Hyun Jin, Sun Il Kim, Chun Kee Chung

**Affiliations:** 1 Interdisciplinary Program in Cognitive Science, Seoul National University College of Humanities, Seoul, Korea; 2 MEG Center, Department of Neurosurgery, Seoul National University Hospital, Seoul, Korea; 3 Department of Statistics, Seoul National University College of Natural Sciences, Seoul, Korea; 4 Department of Neurosurgery, Seoul National University College of Medicine, Seoul, Korea; 5 Department of Biomedical Engineering, Hanyang University, Seoul, Korea; University of Southern California, United States of America

## Abstract

**Background:**

The issue of how differences in timbre are represented in the neural response still has not been well addressed, particularly with regard to the relevant brain mechanisms. Here we employ phasing and clipping of tones to produce auditory stimuli differing to describe the multidimensional nature of timbre. We investigated the auditory response and sensory gating as well, using by magnetoencephalography (MEG).

**Methodology/Principal Findings:**

Thirty-five healthy subjects without hearing deficit participated in the experiments. Two different or same tones in timbre were presented through conditioning (S1) – testing (S2) paradigm as a pair with an interval of 500 ms. As a result, the magnitudes of auditory M50 and M100 responses were different with timbre in both hemispheres. This result might support that timbre, at least by phasing and clipping, is discriminated in the auditory early processing. The second response in a pair affected by S1 in the consecutive stimuli occurred in M100 of the left hemisphere, whereas both M50 and M100 responses to S2 only in the right hemisphere reflected whether two stimuli in a pair were the same or not. Both M50 and M100 magnitudes were different with the presenting order (S1 vs. S2) for both same and different conditions in the both hemispheres.

**Conclusions/Significances:**

Our results demonstrate that the auditory response depends on timbre characteristics. Moreover, it was revealed that the auditory sensory gating is determined not by the stimulus that directly evokes the response, but rather by whether or not the two stimuli are identical in timbre.

## Introduction

Considering the ubiquitous bunch of complex sounds, the ability to detect differences in sound seems to be indispensable. Therefore, studies that reveal which feature of sound people differentiate, how people hear it, and when it is processed provide an important clue about auditory perception in the brain. In research on tonotopic organization, it has been revealed that brain responses correspond sequentially to the height of the frequencies, like retinotopy in vision science [1], [2]. In imaging studies, it has also been shown that the multidimensional aspect of sound is processed by lateralized spectro-temporal analyses in the brain [3], [4], [5], [6]. However, research on the perception of timbre, especially in terms of how neurons in the brain process the timbre perception, has not been addressed (See detail in [7]).

The Acoustical Society of America defines timbre as the attribute of auditory sensation that enables a listener to judge that two non-identical sounds, similarly presented and having the same loudness and pitch, are dissimilar [8]. Thus, timbre should be considered as a trait that describes the multidimensional attribute of sound and that includes changes in the frequency spectrum and in the temporal fluctuation as well [9], [10]. However, previous studies on timbre have been limited to the extraction of fragmentary features of timbre [11], [12], [13]. Some studies employing speech-like stimuli [14], [15], [16] have also provided limited information about timbre perception because they tried to describe the contrasts in frequencies like the qualitative differences in syllables.

In the present study, we set forth to describe the quantitative contrasts of the spectro-temporal properties of multidimensional timbre stimuli. Our goal was to reveal the brain mechanism of timbre discrimination by examining magnetoencephalography (MEG) signals in response to the timbre change. MEG is suitable for overcoming the methodological limitations of functional magnetic resonance imaging (fMRI), such as low temporal resolution and the influence of the noisy environment from surrounding devices. First, we assumed that the subtle differences, which describe the multidimensional properties of timbre, are reflected in the behavioral responses. We could examine the timbre differences in the brain response only if we distinguished these differences in timbre behaviorally. In other words, we could not conclude anything about the differences in brain responses to the differences in timbre, of which we cannot discriminate the difference. Second, we expected distinctive brain responses to the different timbre stimuli. If our brain can discriminate the physical properties of a sound, the brain responses are also distinctive to each stimulus in timbre. Finally, the last issue to be addressed was whether the differences in timbre are perceived when two consecutive tones are delivered, and if so, when and how they are processed in the brain.

## Results

### Synthesizing Spectro-temporal Timbre Stimuli

**Figure 1:**
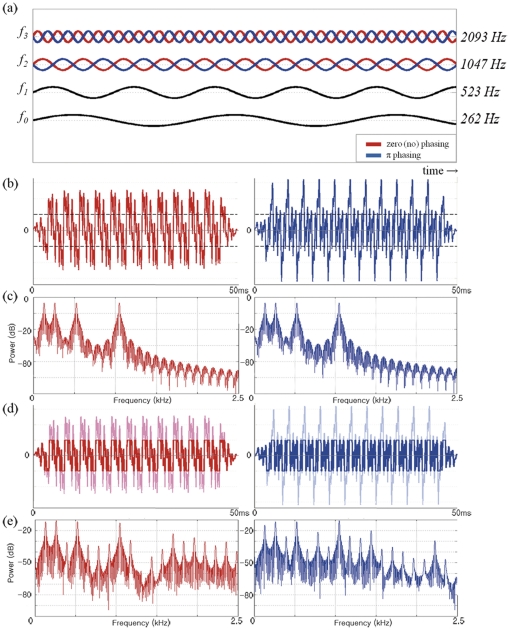
Spectro-temporal aspect of timbre. (**a**) Four frequencies were mixed with phase modulation. Zero (red, left panels in b–e) or π (blue, right panels in b–e) phasing was only applied to two higher frequencies (*f_2_* and *f_3_*). (**b**), (**c**) Waveforms and Fourier analysis of two tones applied with phasing; two tones have different envelopes with same frequency components. (**d**), (**e**) Modulation by clipping the amplitude to the magnitude of a single tone. Two mixtures have similar envelopes but different frequency distribution.

To overcome the limitation of previous studies [11], [13], [15], [17], [18], [19], which failed to describe the multidimensional nature of timbre, we combined frequencies with spectro-temporal differences using the synthesizing techniques of phasing and clipping. First, we combined four frequencies of the same amplitude with or without a phase shift of π for the highest two frequencies in order to generate a temporal difference by phase. Then, we clipped the amplitude of the tone mixture at a single frequency in order to produce a spectral distortion of the sound. By employing phasing and clipping, two different mixed tones having the same frequency components with uniform amplitudes are heard differently, even though their envelopes are similar (Figure 1). In this way, the multidimensional spectro-temporal properties of a timbre could be implemented while keeping the same pitch and loudness.

### Behavioral Responses

We investigated the relationships between score, click, and response time of the behavioral responses. As a result of a Pearson's product-moment correlation analysis, there were no significant correlations between score, click, and response time (See Table 1). The same (t = 9.098, d.f. = 34, P<0.0001), different (t = 6.053, d.f. = 34, P<0.0001), and total (t = 10.029, d.f. = 34, P<0.0001) scores were significantly above chance level (50%) as determined by a one-sample t-test (two-tailed) with a test value of 50, even though there was a difference (paired t-test, t = −2.043, d.f. = 34, P = 0.049, two-tailed) between the same and different scores (See Descriptive Statistics in Table 2).

**Table 1:** Correlations between *Score*, *Click*, and *Response Time.*

	ρ	P-value
*Click-Score*	.051	.7716
*Response Time-Score*	-.276	.1086
*Click-Response Time*	.079	.6519

Statistics: Pearson's product-moment correlation (two-tailed).

No correlation between *Score*, *Click*, and *Response Time*.

**Table 2:** Descriptive Statistics of *Score*, *Response Time*, *Click*, Same and Different *Score.*

Variables	N	Range	Min.	Max.	Mean	SD
Score (%)	35	46.0	48.0	94.0	69.31	11.39
Response Time (ms)	35	1351.0	460.0	1811.0	995.40	266.67
Click (times)	35	47.0	27.0	74.0	46.89	9.70
Same (%)	35	60.00	38.00	98.00	72.43	14.59
Different (%)	35	58.00	34.00	92.00	65.74	15.39

### Brain Responses

**Figure 2:**
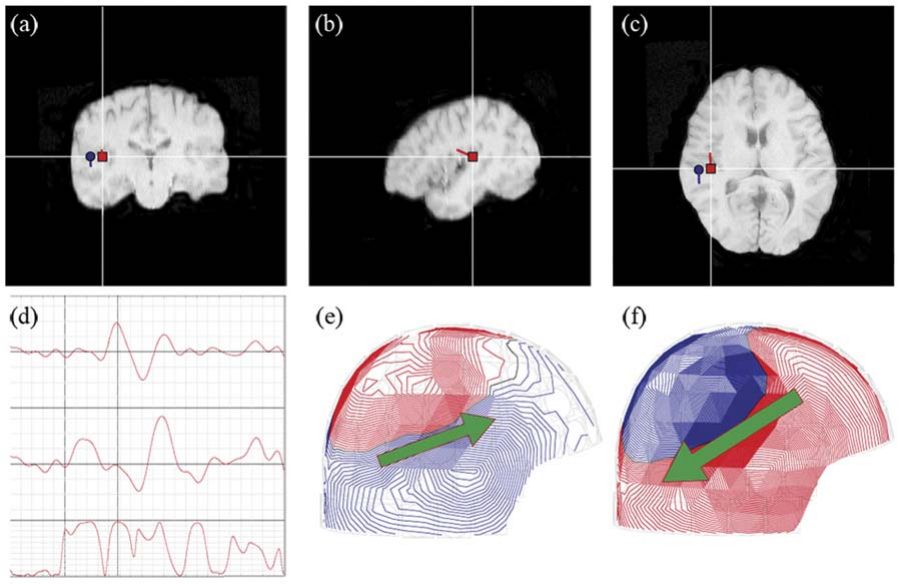
Equivalent current dipoles of the auditory brain response. **(a) Coronal, (b) sagittal, and (c) axial views of the dipole localization rendered on TR images of a subject.** The blue dot indicates the M100 dipole, while the red rectangle indicates the M50 dipole. The locations of the two dipoles are localized in the primary auditory cortex. **(d) Source waveform of M50 and M100 dipoles.** The waveforms of the M100 (in the upper), M50 (in the middle) dipoles, and goodness-of-fit of the two dipoles (in the bottom). A left vertical line indicates the stimulus onset time (t0), whereas a right vertical line corresponds to the time at which M50 dipole is fitted. **The topographies of the magnetic fields of (e) M50 and (f) M100.** The locations of two dipoles of M50 and M100 are similar, although the orientations of two dipoles are the opposite.

The M50 response was colocated with that of the M100 [20], [21], [22]. There were no differences related to the location and latency of any comparisons of interest, including *condition* and *presenting order* (Figure 2).

The question was whether the early responses represented as M50 and M100 (the magnetic counterparts of the electrophysiological responses P50 and N100, respectively) in the auditory cortex reflect the timbre differences of the stimuli. If so, this would provide a window on the neural events underlying the perceptual discrimination of timbre.

**Figure 3:**
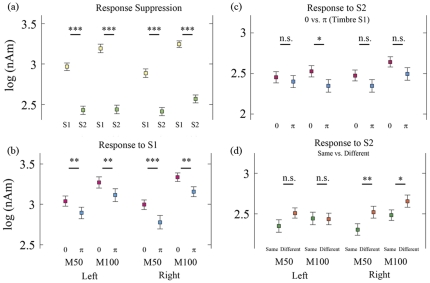
Comparison of the dipole strengths. (**a**) Response suppression. Dipole strengths of the responses to S1 (yellow) vs. S2 (bright green). N = 35 (subjects)×2 (sessions)×2 (conditions). (**b**) Dipole strengths of the S1 responses to S1 stimuli of 0 phase modulation vs. π. Dipole strengths of 0 phase (magenta) is significantly higher than those of π phase (cyan). N = 35 (subjects)×2 (sessions). (**c**) Dipole strengths of the S2 responses to S1 stimuli of 0 phase modulation vs. π. Only M100s of the left hemisphere were significantly different. N = 35 (subjects)×2 (sessions). (**d**) Dipole strengths of the S2 responses to stimuli in same pairs vs. different. In the right hemisphere, M50 and M100 in different conditions were significantly higher than those in the same condition. N = 35 (subjects)×2 (sessions). For all, the error bar indicates standard error of mean (SEM). All values are logarithmically transformed. N: number of independent data points. *: significant at the 0.05 level, **: significant at the 0.01 level, ***: significant at the 0.001 level, n.s.: not significant.

Indeed, our results support such a discrimination; the responses to S1 reflected the timbre differences [comparison variable (*timbre of S1*: 0 vs. π, F_(1, 34)_ = 24.32, P<0.0001), See Table 3 and Figure 3b]. Furthermore, this timbre discrimination was distinguished by *components* (M50 vs. M100: F_(1, 34)_ = 40.63, P<0.0001), which may indicate the presence of different neural sources for M50 and M100. There was no difference between *hemispheres* (Left vs. Right: F_(1, 34)_ = 0.02, P = 0.8806, not significant). Upon scrutinizing the data by dividing them into 4 groups by *hemispheres* and *components*, there were significant differences between timbre of S1 in all M50 and M100 components for both hemispheres (Left M50: F_(1, 34)_ = 4.71, P = 0.0371; Left M100: F_(1, 34)_ = 8.65, P = 0.0058; Right M50: F_(1, 34)_ = 6.02, P = 0.0194; Right M100: F_(1, 34)_ = 14.6, P = 0.0005; See Table 3). These data imply that early auditory processing near 50 ms and 100 ms is involved in distinguishing the timbre of stimuli in both hemispheres.

**Table 3:** Auditory M50/M100 Responses to S1.

Variables^1^			LT/M50		LT/M100		RT/M50		RT/M100	
	F	P-value	F	P-value	F	P-value	F	P-value	F	P-value
timbre_S1	24.32	<.0001***	4.71	0.0371*	8.65	0.0058**	6.02	0.0194**	14.6	0.0005***
hemisphere	0.02	0.8806								
component	40.63	<.0001***								
timbre_S1 × hemisphere	0.18	0.6737								
timbre_S1 × component	0	0.9728								
hemisphere × component	1	0.3256								
timbre_S1 × hemisphere × component	0.06	0.8082								

Statistics: Repeated measures using linear mixed model.

Dependent variable: Dipole Strength Q of response to S1 (logarithmically transformed).

Independent variables: timbre_S1, hemisphere, component.

Degree of freedom of numerator  = 1, Degree of freedom of numerator  = 34.

Covariance structures: heterogeneous Toeplitz.

*P<0.05,

**P<0.01,

***P<0.001.

The next question was whether the response to S2 is determined solely by S2 irrespective of S1 or by the discrepancy of stimuli in a pair. If the auditory response is only affected by the most recent stimulus, then only a feed-forward mechanism exists at this early stage of auditory processing, and the response produced by S2 should depend only on the timbre of S2, regardless of the timbre of S1. Otherwise, if the response is influenced by the preceding stimulus, a feedback comparison of discrepancy, as well as the timbre discrimination of a single tone, should be processed. Our result, which was tested by a repeated measures analysis using a linear mixed model [comparison variables (*timbre of S1*: 0 or π, *timbre of S2*: 0 or π)], indicated that the response to S2 was not determined by S2 (F_(1, 34)_ = 0.15, P = 0.6981; See Table 4 and Figure 3c), but by S1 (F_(1, 34)_ = 6.33, P = 0.0167). Moreover, the response to S2 was modulated by the equality of stimuli in a pair (F_(1, 34)_ = 11.59, P = 0.0017; See Figure 3d). Dividing the data into *hemispheres* and *components*, we found that, for M100, this influence of S1 was only valid for the left hemisphere (F_(1, 34)_ = 4.94, P = 0.0330; Table 4). In contrast, by serial comparison, the timbre differences were revealed in both the right M50 (F_(1, 34)_ = 10.32, P = 0.0029) and the right M100 (F_(1, 34)_ = 5.96, P = 0.0200).

**Table 4:** Auditory M50/M100 Responses to S2.

Variables^1^			LT/M50		LT/M100		RT/M50		RT/M100	
	F	P-value	F	P-value	F	P-value	F	P-value	F	P-value
timbre_S1	6.33	0.0167*	0.39	0.5346	4.94	0.033*	2.88	0.0987	2.75	0.1065
timbre_S2	0.15	0.6981	0.01	0.9109	2.83	0.1019	0.12	0.7366	0.85	0.3629
hemisphere	1.11	0.2987								
component	2.84	0.1013								
timbre_S1 × timbre_S2	11.59	0.0017**	3.53	0.0689	0.01	0.9432	10.32	0.0029**	5.96	0.02*
hemisphere × timbre_S1	0.06	0.8041								
component × timbre_S1	0.96	0.3344								
hemisphere × timbre_S2	1.04	0.3149								
component × timbre_S2	0.07	0.7891								
hemisphere × component	1.85	0.1829								
hemisphere × timbre_S1 × timbre_S2	2.11	0.1552								
component × timbre_S1 × timbre_S2	2.62	0.115								
hemisphere × component × timbre_S2	2.78	0.1047								
hemisphere × component × timbre_S1	0.49	0.4878								
hemisphere × component × timbre_S1 × timbre_S2	0.82	0.3708								

Statistics: Repeated measures using linear mixed model.

Dependent variable: Dipole Strength Q of response to S2 (logarithmically transformed).

1 Independent variables: timbre_S1, timbre_S2, hemisphere, component.

Degree of freedom of numerator  = 1, Degree of freedom of numerator  = 34.

Covariance structures: heterogeneous Toeplitz.

*P<0.05,

**P<0.01.

We also observed a response suppression of the second of two consecutive stimuli (i.e., the gating effect), which is in line with previous studies [23], [24], [25], [26], [27], [28]. The response difference by *presenting order,* S1 vs. S2, was strongly significant for all *components* in both *hemispheres* (F_(1, 34)_ = 341.6, P<0.0001; See Table 5 and Figure 3a). These effects were also confirmed when the data were divided across both *hemispheres* and *components* (Left M50: F_(1, 34)_ = 98.9, P<0.0001; Left M100: F_(1, 34)_ = 107.59, P<0.0001; Right M50: F_(1, 34)_ = 87.96, P<0.0001; Right M100: F_(1, 34)_ = 103.99, P<0.0001). Moreover, there was an interaction between *presenting order* and *condition* (same vs. different: F_(1, 34)_ = 17.32, P = 0.0002), whereas no main effect of *condition* was found (F_(1, 34)_ = 1.42, P = 0.2414, not significant). This indicates that the gating effect could be separated into **gating in** and **out** by the equality of stimuli. Furthermore, these gating differences were observed in the left M50 (F_(1, 34)_ = 6.52, P = 0.0153), the right M100 (F_(1, 34)_ = 20.41, P<0.0001), and the right M50 (F_(1, 34)_ = 10.78, P = 0.0024), but not in the left M100 (F_(1, 34)_ = 1.85, P = 0.1826, not significant). This separation into **gating in** and **out** also indicates the discrimination (of timbre) by serial comparison. These findings are consistent with our results for S2, which is the serial comparison in the right hemisphere.

**Table 5:** Effect by presenting order and condition: Gating in and out effect.

Variables^1^			LT/M50		LT/M100		RT/M50		RT/M100	
	F	P-value	F	P-value	F	P-value	F	P-value	F	P-value
order	341.6	<.0001***	98.9	<.0001***	107.59	<.0001***	87.96	<.0001***	103.99	<.0001***
condition	1.42	0.2414	0.65	0.4269	0.7	0.4083	2.84	0.1009	3.71	0.0626
hemisphere	0.58	0.4532								
component	22.11	<.0001***								
order × condition	17.32	0.0002***	6.52	0.0153*	1.85	0.1826	10.78	0.0024**	20.41	<.0001***
order × hemisphere	1.6	0.214								
order × component	8.93	0.0052**								
condition × hemisphere	2.32	0.1372								
condition × component	2.17	0.1498								
hemisphere × component	1.75	0.1942								
order × condition × hemisphere	2.41	0.1296								
order × condition × component	1.23	0.2752								
condition × hemisphere × component	1.53	0.2252								
order × hemisphere × component	0.33	0.5694								
order × condition × hemisphere × component	2.01	0.1656								

Statistics: Repeated measures using linear mixed model.

Dependent variable: Dipole Strength Q of response (logarithmically transformed).

1 Independent variables: order, condition, hemisphere, component.

Covariance structures: heterogeneous Toeplitz.

*P<0.05,

**P<0.01,

***P<0.001.

## Discussion

First, we introduced the concept of creating stimuli that describe well the spectro-temporal subtle changes in timbre. Timbre is conceptually determined by the residual definition that excludes the defined attributes so that it seems to be complicated to describe the characteristics of timbre itself in order to make experimental contrasts. This is why many scientists have used musical instruments or sinusoidal mixtures of different frequencies that have different envelopes in their experimental designs [11], [13], [14], [15], [16] because these stimuli have explicit contrasts in timbre without having to describe their attributes of contrast. Our methods to create stimuli contributed not only to the description of these stimuli, as in previous studies, but also provided a template by which the contrast in timbre can be expressed. Moreover, we can directly apply these stimuli to describe the characteristics of speech-like stimuli in many experiments, since our stimuli have four different frequencies, which is the number of formants of human voices.

Our results were derived from the brain responses in cases of correct discrimination, which were based on the behavioral results. In other words, the present study assumed that the correct behavior was conducted from the correct perception. Therefore, we cannot explain the cases in which the behavioral judgment failed in our experiment. Nevertheless, our hypothesis that the differences in timbre are affected by the perception, and the result that these differences in the perception level are reflected in the behavioral and brain response, were sufficiently supported by our results. However, a perceptual failure affected by the incorrect decision can be considered part of the error-making system in the cognitive decision-making process in the perspective of the top-down processing of the perception. Moreover, our results may be strongly supported by timbre discrimination during passive listening without any required task and by the elimination of the confusion caused by the physical aspects and the psychological ones when using a roving paradigm [29], for example.

With the comparison of the responses to single tones (S1), we confirmed that the differences in timbre by 0 and π phase modulation were represented by the strength differences in the responses near the auditory cortex and within 50 ms and 100 ms after stimuli delivery. In addition, based on the finding that the strengths of the 0-phase were consistently larger than those of the π-phase, timbre induced by the differences in phase of stimuli was consistently reflected in the brain responses. This means that the differences in timbre were already affected at the perception level. Then, why are the 0-phase responses larger than those of π-phase? The dipole source estimated from MEG signals is assumed to be the current source from the synchronization of thousands of neural activities [30]. Based on this assumption, our results can be explained as follows: the 0-phase modulation indicates that the harmonics of input frequencies were temporally synchronized, and so they may induce stronger synchronization of the neural activities. In contrast, the harmonics in π-phase modulation were perceived with a temporal gap, so that the neural activities were less synchronized. Moreover, there were differences in the brain responses between M50 and M100 but no difference between hemispheres. These findings suggest that the differences in stimuli directly affect the brain responses in terms of the feed-forward mechanism and also that the M50 and M100 play different functional roles in auditory processing [31], [32]. In agreement with previous studies that showed comprehensive convergence of enhanced magnitudes of M50 in children in developmental studies [33], [34] and the susceptibility of M50 to the physical plenitude of stimuli [35], our results suggest that subtle changes of timbre stimuli are reflected in the brain response within 50 ms.

From the results of the consecutive stimuli, the feedback system of perception, as well as the feed-forward mechanism in single tone processing, can be explained. The second response affected by S1 in the consecutive stimuli occurred in M100 of the left hemisphere. Previous studies have pointed out that the spectral analysis of auditory processing occurs near 200 ms in the right hemisphere [13], [18]. However, the differences elicited by the stimuli in their studies were also seen in M100 of the left hemisphere. Moreover, the M100 responses in the left hemisphere seemed to be stronger than those in the right hemisphere [36]. These results may be interpreted that the temporal range of the functional role of the left M100 was wide so that the influence of S1 was retained [37]. This is the feedback mechanism by which the effects of S1 responses persisted to the perception of S2 stimuli. In contrast, the fact that both M50 and M100 responses to S2 only in the right hemisphere reflected whether two stimuli in a pair were the same or not may be translated into the continuous monitoring of auditory comparison processes [32]. For the final outcome, the differences in hemispheres and in M50 and M100 components in this study can help to explain the asymmetric roles, which are in line with previous studies [3], [5], [38]. It seems that the left hemisphere tends to dominate in temporal aspects of auditory perception, while the right hemisphere is responsible for the comparison of the elements of stimuli by analyzing spectro-temporal attributes of timbre.

We also showed that a gating effect, by which the second response to repetitive stimuli is attenuated, depended on whether two consecutive tones were the same or not. Our results suggest that the gating effect is not caused by suppression by the habituation to the repetitive stimuli but by the filter of the comparison with the prior stimulus. Moreover, the laterality of the gating effect in the right hemisphere agrees with our results above, which is the spectral comparison of the repetitive stimuli occurs in the right hemisphere. Indeed, the gating effect is also a concomitant phenomenon at the early auditory perception.

Here, we showed that the human ability to discriminate the subtle timbre changes of auditory stimuli is processed at very early stages, near 50 ms, in the auditory perception, and the consequences from the discrimination processing are clearly reflected in the brain responses in the auditory cortex. Our results may provide links between timbre discrimination and interpretation [39], which encompass the functional routes from auditory perception to cognition [40].

## Materials and Methods

### Participants

Forty-two healthy volunteers were recruited by means of a public announcement; five were excluded by our experimental criteria of age, handedness, and pathological history. The 37 remaining subjects (age, 26.0±3.5 years, mean ± SD; 15 males) who participated in the experiment had normal hearing and were right-handed according to the Edinburgh Handedness Inventory [41] (89.5±13.6). This study was approved by the Institutional Review Board of the Clinical Research Institute, Seoul National University Hospital, and written informed consent was obtained from all subjects before proceeding with the measurements, in accordance with the regulations of the Institutional Review Board of the Clinical Research Institute, Seoul National University Hospital, which were based on the principles expressed in the Declaration of Helsinki (IRB No. C-1003-015-311).

### Stimulus Preparation and Presentation

The auditory stimuli consist of four sinusoidal signals whose frequencies were 262, 523, 1047, and 2093 Hz, which corresponded to the musical notes C4, C5, C6, and C7, respectively. Two different synthesizing (signal processing) methods were applied. First, two higher frequencies (1047 and 2093 Hz) were shifted in phase by π in order to emphasize the effect of phase shifting according to the following simple equation:
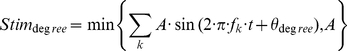
where *t* is the duration of a mixture tone, *k* is the index of harmonic tones from 0 to 3, i.e., *f_k_* is the k^th^ frequency component, and *θ _degree_* is the degree of phase shifting in two higher frequencies, *f_2_* and *f_3_*. So, *θ _degree_* is 0 or π. The duration of each tone mixture was 50 ms, including 5 ms of rise and fall time. Then, the two-tone mixtures were clipped at the magnitude of the single pure tones making up the mixture. These stimuli were generated by using ordinary signal processing in MATLAB™ 7 (The Mathworks, Inc., Natick, MA, USA). The sampling rate of the auditory streaming output was 44100 Hz with 16 bits of resolution. Inter-pair intervals varied between 5.5 and 6.5 s (mean, 6 s). The auditory stimuli were binaurally presented at 100 dB SPL via Stim2™ (Neuroscan, El Paso, TX, USA) using plastic tubes of 50-cm length and silicone earpieces. A silent movie clip (Love Actually, 2003, Universal Pictures, USA) was presented by a video projector from outside of the shielded room in order to retain arousal during measurements [42], since the target responses, M50 and M100, are not affected by variations in alertness, except in extreme cases, such as with sleep [43].

### Procedures

**Figure 4:**
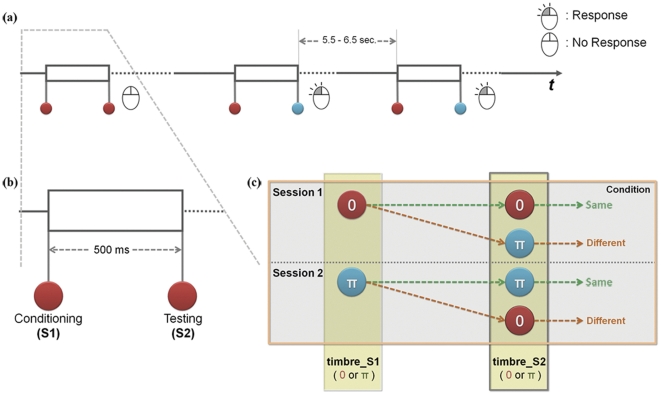
Schematic diagram of Experimental Procedure. (**a**) Auditory stimuli were presented as a pair with 5.5–6.5 s (mean, 6 s). A pair consists of two identical tones (same pairs) or two different tones in timbre (different pairs). Participants were asked to detect the different pairs. (**b**) Two consecutive tones were separated with 500 ms intervals. (**c**) Each session was comprised of 50 same pairs and 50 different pairs. Two sessions were counterbalanced by interchanging the S1 stimulus.

This experiment is based on the conditioning-testing paradigm, in which the auditory stimuli are presented as a pair separated by a certain time interval. This paradigm has typically been used to estimate the pre-attentive effect on the gating deficit in schizophrenia with one simple tone such as a click or pip sound [27]. We modified this paradigm by using two tones that were identical or that differed in timbre, so that we could estimate response suppression with repeated identical stimuli, as well as the difference in response suppression resulting from stimulus pairs that differed in timbre. A pair was comprised of two identical ('same pairs') or different tones ('different pairs') separated by an onset-interval of 500 ms. Participants were asked to click a mouse button whenever they heard a different pair. The experiment had two counterbalanced sessions in which 50 same and 50 different pairs were delivered pseudo-randomly; the S1 tone in one session was used as an S2 tone of the different pairs in another session. (See Figure 4).

### Magnetoencephalography Measurement

Electromagnetic brain activities evoked by auditory stimuli were acquired using a 306-channel whole-head MEG System (VectorView, Elekta Neuromag Oy, Helsinki, Finland), which was comprised of 102 identical triple sensor elements in a magnetically shielded room. Each sensor element consisted of two orthogonal planar gradiometers and one magnetometer coupled to a multi-Superconducting Quantum Interference Device (SQUID) and provided three independent measurements of the magnetic fields. The EOG was acquired in order to eliminate eye-movement artifacts. Signals were analog-filtered between 0.1 and 200 Hz at a sampling frequency of 1000 Hz. Head movements were tracked with four additional head position indicator coils attached to the participants' heads. For removing magnetoencephalographic artifacts, the temporal signal space separation (tSSS) method implemented by Maxfilter™ Software (Elekta Neuromag Oy, Helsinki, Finland) was used [44].

### Data Preprocessing and Analysis

We excluded two subjects, who clicked in less than a quarter or more than three quarters of the trial, from further analysis.

MEG signals were digitally filtered using a band-pass filter between 5 and 30 Hz. Epochs with a duration of 500 ms were extracted for each tone stimulus, beginning 100 ms before stimulus onset. Epochs for which the MEG signals exceeded 2000 fT/cm (for gradiometers) or 4000 fT (for magnetometers) and for which the EOG signal exceeded 80 µV were excluded from offline averaging. Also, we excluded both epochs of pairs for which the participant failed in the behavioral timbre detection in order to prevent incorrect answers from contaminating the responses to the correct answers. Baseline correction between −100 and 0 ms was performed after averaging. All preprocessing was executed using MNE Suite (version 2.7, Martinos Center for Biomedical Imaging, Charlestown, MA, USA). Equivalent current dipoles (ECD) were extracted for conditioning (S1) and testing (S2) along with presenting order, on both hemispheres, and in both same and different conditions, respectively using Neuromag™ software. In order to localize the dipole, we applied the spherical model. Several studies have reported the location of the M50 dipole, and it is known to be colocated with the M100 dipole [20]. Therefore, our strategy was to fit M100 dipoles as reference points, and then to localize the M50 dipole in reference to a nearby location with the opposite magnetic topo-field. M50 and M100 dipoles were identified as the maximum peak of the brain activity in auditory cortex between 40 and 80 ms for M50, and between 80 and 150 ms for M100. For each dipole, three-dimensional locations, latency, and dipole strength were statistically considered as dependent variables.

### Statistical Analysis

For statistical analysis, we tested whether all variables followed a normal distribution using the Kolmogorov-Smirnov test, and confirmed the analysis with p-p plots. If necessary, we transformed the variables into logarithmic scales. The evoked responses were estimated based on the averaged magnetic field, which can be thought of as a statistics from a series of physiological events [45]. Moreover, there might exist individual variances of the brain responses caused by gender, age or any else [33]. Although the electro-magnetic signals from human brain have common features across all individuals, the magnitude of signal varies with each individual; unobservable individual variances should be considered. The linear mixed model (LMM) concerns the parameter of fixed and the unobservable random effects as well. Moreover, it can allow for both correlation and heterogeneous variances, and therefore, it has flexibility in modeling the covariance structure. Then, we applied a repeated measures analysis using linear mixed models as following:
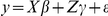
where y is vector of observations; X and Z are matrices of regressors of β and γ, respectively; β is vector of fixed effects, which represent the effects of timbre of S1, timbre of S2, order, condition, hemisphere or component along the statistical inferences; γ is vector of independent and identically-distributed (IID) random effects which represent the inter-subject variability with variance-covariance matrix var (γ) = G; ε is the residual random error term in the model and variance var (ε) = R. The variance of y is thus 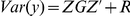



The model matrix Z is set up in the same fashion as X, the model matrix for the fixed-effects parameters. For G and R, we can select any covariance structure which can explain the data. The model parameters were estimated by the maximum likelihood-base method and considered significant if the P values were <0.05, <0.01 and <0.001 respectively. To obtain the estimates of β and γ, the mixed model equation was used as the standard method. The statistical inferences were obtained by testing the null hypothesis (H_0_) in which the linear combination of the estimated parameters, fixed and random effects, which are β and γ respectively, are all zeros.
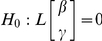



To estimate the random effect, we assumed the heterogeneous Toeplitz model as the covariance structure, based on the Akaike's information criterion (AIC) and Bayesian information criterion (BIC) value, among different covariance structures. For all data sets, the sample size was 35.
